# JOINT TRAJECTORIES OF CAREGIVING BURDEN AND BENEFITS AMONG FAMILY CAREGIVERS OF OLDER ADULTS

**DOI:** 10.1093/geroni/igae098.0642

**Published:** 2024-12-31

**Authors:** Yongjing Ping, Jeremy Lim-Soh, Truls Østbye, Rahul Malhotra

**Affiliations:** Duke-NUS Medical School, Singapore, Singapore; Duke-NUS Medical School, Singapore, Singapore; Duke University, Durham, North Carolina, United States; Duke-NUS Medical School, Singapore, Singapore

## Abstract

Caregiving is often associated with burden, but it can also benefit family caregivers. While the two phenomena can co-exist, existing studies have largely considered the two separately. Studies considering the two together are mostly cross-sectional, even though caregiving is dynamic over time, requiring longitudinal study. It is also important to understand the heterogeneity in the joint (co-existing) trajectories (patterns over time) of caregiving burden and benefits as it can help identify family caregivers who are continuously vulnerable and determine modifiable factors linked with detrimental trajectories. We conducted group-based multi-trajectory modelling on longitudinal data – collected at four time-points, at six-to-twelve-month intervals – on burden (negative domains of modified Caregiver Reaction Assessment) and benefits (short Positive Aspects of Caregiving scale) from 274 family caregivers in Singapore. We identified four trajectory groups, each with distinct joint trajectories of burden and benefits – “balanced” (44.5%, least burden and relatively high benefits), “satisfied” (23.0%, second lowest burden and highest benefits), “dissatisfied” (19.0%, second highest burden and least benefits), and “intensive” (13.5%, highest burden and relatively high benefits). Relative to caregivers in the “balanced” group, those supported by trained migrant domestic workers were less likely to be in the other three groups and those more prepared for caregiving were more likely to be in the “satisfied” group. The findings highlight the heterogeneity in the caregiving experience over time and suggest that promoting migrant domestic worker training and family caregiver preparedness for caregiving may contribute to advantageous patterns of burden and benefits of caregiving over time.

